# Surgical Outcome of Trigeminal Schwannomas

**DOI:** 10.3390/cancers13061310

**Published:** 2021-03-15

**Authors:** Amir Kaywan Aftahy, Maximilian Groll, Melanie Barz, Arthur Wagner, Nicole Lange, Vicki Marie Butenschön, Claire Delbridge, Denise Bernhardt, Bernhard Meyer, Chiara Negwer, Jens Gempt

**Affiliations:** 1 Department of Neurosurgery, Klinikum rechts der Isar, School of Medicine, Technical University Munich, 81675 Munich, Germany; maxi.groll@gmx.de (M.G.); Melanie.barz@tum.de (M.B.); arthur.wagner@tum.de (A.W.); nicole.lange@tum.de (N.L.); vicki.butenschoen@tum.de (V.M.B.); Bernhard.Meyer@tum.de (B.M.); Chiara.Negwer@tum.de (C.N.); jens.gempt@tum.de (J.G.); 2 Department of Neuropathology, Institute of Pathology, Klinikum rechts der Isar, School of Medicine, Technical University Munich, 81675 Munich, Germany; c.delbridge@tum.de; 3 Department of Radiation Oncology, Klinikum rechts der Isar, School of Medicine, Technical University Munich, 81675 Munich, Germany; Denise.Bernhardt@mri.tum.de

**Keywords:** schwannoma, trigeminal nerve, skull base, operative technique, neurosurgical oncology

## Abstract

**Simple Summary:**

Trigeminal schwannomas are the most common among non-vestibular schwannomas. Treatment of trigeminal schwannomas may be challenging due to critical anatomical relations and involvement of different aspects of the skull base. Advances in microsurgery have led to improved outcomes over the last decades, whereas in contrast, some advocate stereotactic radiotherapy as an effective therapy, controlling the tumor volume with few complications. In this manuscript, we present outcome and adverse events in a contemporary cohort of trigeminal schwannomas and discuss surgical advantages and disadvantages of different performed classic skull-base approaches.

**Abstract:**

(1) Background: As resection of trigeminal schwannomas is challenging, due to anatomical involvement of the anterior, middle and posterior fossa, the appropriate approach is important. We report our experience with surgical resection of trigeminal schwannomas by simple and classic skull-base approaches. (2) Methods: We performed a retrospective single-center study including patients who underwent surgery for trigeminal schwannoma tumors between June 2007 and May 2020, concentrating on surgical technique, extent of resection, postoperative outcome and complications. (3) Results: We included 13 patients (median age 57.5 with range of 36-83 years, 53.8% (7/13) female. The most common preoperative clinical presentations were facial pain in six (46.2%), hypoacusis in four (30.8%), trigeminal nerve hypesthesia in 11 (V1 46.2% (6/13), V2 (61.5% (8/13), V3 46.2% (6/13)) and headache in three (23.1%) patients. In three cases, the tumor was resected in a two-stage technique. The intradural subtemporal approach was performed in six cases, the extradural technique in two cases, the retrosigmoid approach in five cases, a Kawase approach in two cases and the transnasal endoscopic approach in one case. The gross total resection rate was 76.9% (10/13) and the median follow-up time 24.0 (0–136) months. Three (23.1%) patients developed postoperative anesthesia in at least one branch of the trigeminal nerve. Trigeminal motor function was preserved in 11 (84.6%) patients. Ten of the 11 patients (76.9%) who reported decreased gustation, cerebellar ataxia, visual impairment, or headache improved postoperatively. Two (15.4%) patients exhibited minimal facial palsy (House and Brackmann II–III), which resolved during the follow-up. The total adverse event rate requiring surgical intervention during follow-up was 7.7%. Surgery-related mortality was 0%. (4) Conclusions: Trigeminal schwannomas are rare benign lesions with intra- and extracranial extension. Considering the low operative morbidity and satisfying functional outcome, gross total resection of trigeminal schwannomas is achievable by classic, but also individually tailored approaches. More invasive or combined techniques were not needed with meticulous surgical planning.

## 1. Introduction

Trigeminal schwannomas are the most common among non-vestibular schwannomas and account for 1.0 to 8.0% of intracranial schwannomas and 0.1 to 0.5% of intracranial tumors [1,2]. Treatment of trigeminal schwannomas may be challenging due to critical anatomical relations and involvement of different aspects of the skull base. When planning an optimal surgical treatment strategy, one must take various factors into account. 

**Figure 1:**
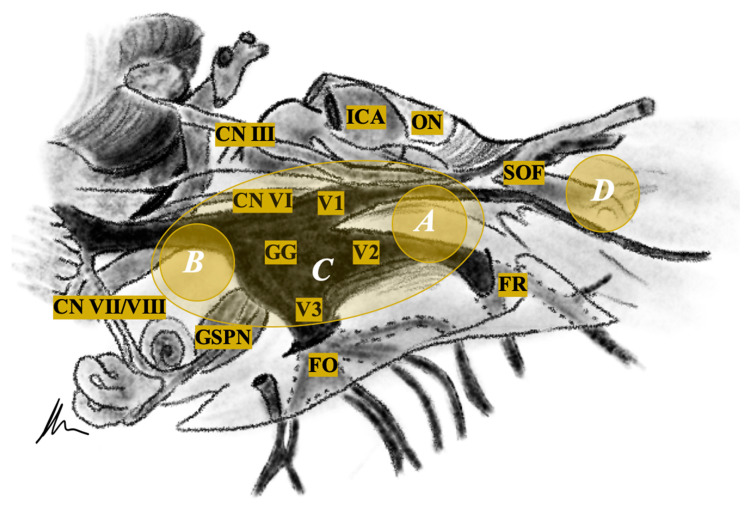
Schematic representation of the trigeminal nerve, its branches, their anatomical localization, adjacent neurovascular structures and tumor classification (white letters). CN = Cranial Nerve, FO = Foramen Ovale, FR = Foramen Rotundum, GG = Gasserian Ganglion, GSPN = Greater Superficial Petrosal Nerve, ICA = Internal Carotid Artery, ON = Optic Nerve, SOF = Superior Orbital Fissure, V1 = Ophthalmic Division, V2 = Maxillary Division, V3 = Mandibular Division.

They can arise anywhere between the root and the distal extracranial branches of the trigeminal nerve originating from the root, the ganglion, or the peripheral branches of the trigeminal nerve [2] (Figure 1). 

**Figure 2:**
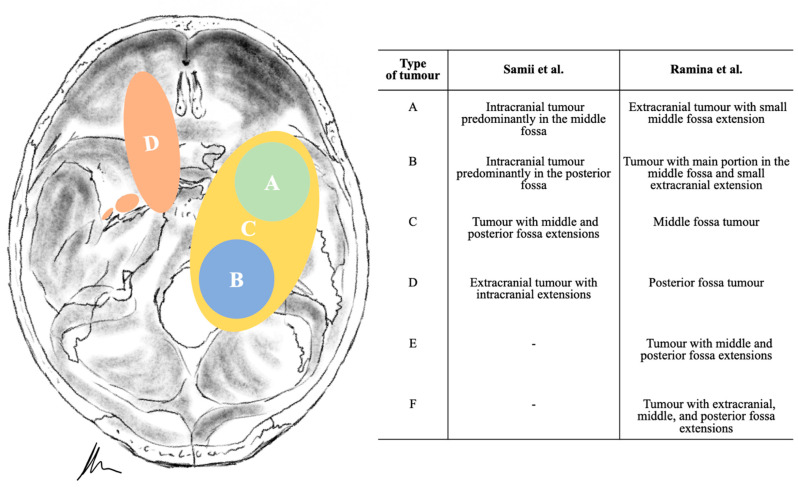
Illustration (according to Samii) and overview of the classification systems of trigeminal schwannomas.

Different classifications have been described in the literature [3,4,5,6,7]. Samii et al. proposed a simplified and useful division into four categories based on radiological findings, whereas Ramina et al. added modifications according to the level of difficulty, describing also two-stage approaches for the most difficult so-called “Type F” tumors [5,6] (Figure 2).

Advances in microsurgery and various skull base approaches have led to improved outcomes and a high rate of complete resection over the last decades, whereas in contrast, some advocate stereotactic radiotherapy as an effective therapy for trigeminal schwannoma, controlling the tumor volume with few complications [1].

To access them, depending on their location and above-mentioned classification, different approaches exist and are required, with various extensions and technical nuances, such as the retrosigmoid approach, different middle fossa approaches and, in some cases and more recently discussed and evolved, the transnasal endoscopic approach [1,4,5,6,8,9,10,11,12].

In this manuscript, we present outcome and adverse events in a contemporary cohort of trigeminal schwannomas and discuss surgical advantages and disadvantages of different performed classic skull-base approaches.

## 2. Material and Methods

### 2.1. Study Design and Outcome Parameters

We performed an observational retrospective single-center study. All patients who underwent surgery for trigeminal schwannomas between June 2007 and May 2020 at the neurosurgical department of the Technical University in Munich, Germany, were included. The clinical records of patients were analyzed according to surgical approach, pre- and postoperative neurological and cranial nerve status and adverse events during follow-up visits. Tumor location was classified according to aforementioned classifications. The extent of resection was defined by comparing pre- and postoperative 3.0 T cranial magnetic resonance imaging (MRI) using T1 ± contrast agent sequences by manual volumetric segmentation, using the Origin^®^ software (Brainlab, version 3.1, Brainlab AG, Munich, Germany).

### 2.2. Statistics 

Statistical analysis was performed using the software STATA (version 13.1, 2011, StataCorp, College Station, TX, USA). Normal distribution was assumed according to the central limit theorem. Data in text and graphs are shown as median (mdn.) with interquartile range (IQR) or mean ± standard deviation (SD). The following *p* values have been considered as significant: * *p* < 0.05, ** *p* < 0.01, *** *p* < 0.001”.

### 2.3. Ethics Approval 

The local ethics committee of the Technical University Munich, School of Medicine (231/20 S-EB) approved our study. We conducted it in accordance with the ethical standards of the 1964 Declaration of Helsinki and its later amendments [13].

The requirement for written informed consent was waived by the ethics committee.

### 2.4. Surgical Approaches

#### 2.4.1. Intra- or Extradural Subtemporal Middle Fossa Approach

**Figure 3:**
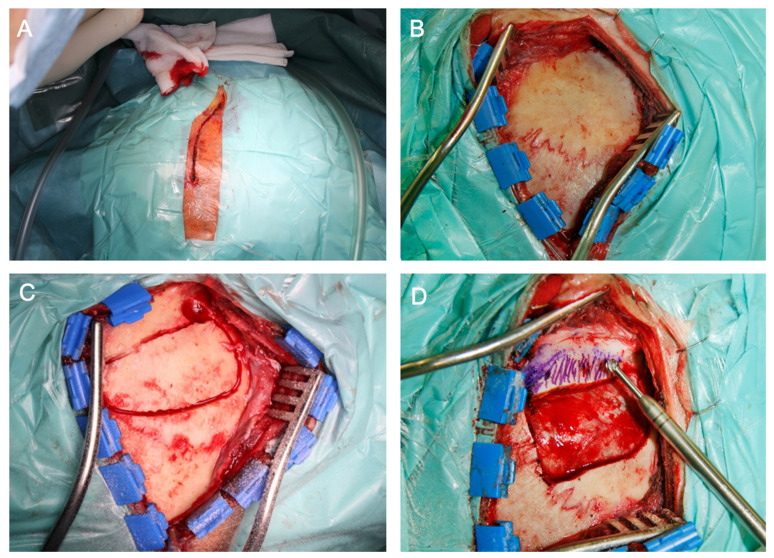
Intraoperative illustration of a subtemporal craniotomy. The patient is placed in a supine position. The head should be rotated as much as possible (~90°) while utilizing a large shoulder roll underneath the ipsilateral shoulder to minimize neck torsion. The head is then tilted ~20° toward the floor for gravity retraction to mobilize the temporal lobe away from the middle fossa. (**A**) A preauricular semi-curvilinear incision is performed, alternatively, a linear incision may be done as well. Large subtemporal lesions benefit from a horseshoe-shaped incision. For lesions that require access to the anterior temporal pole, a small reverse question mark incision would be appropriate, too. (**B**,**C**) After placing a generous single burr hole just above the root of the zygoma, the dura is mobilized away from the inner table of the calvarium. It is essential to avoid early injury to the dura in order to protect the lobe during extradural subtemporal dissection and later petrosectomy. The craniotomy should be done as close to the middle fossa floor as possible by identifying the upper edge of the root of zygoma which marks the level of the middle fossa floor. The floor is oblique and slopes slightly superiorly from the anterior to posterior direction; the inferior edge of the craniotomy should be only slightly above the level of the zygoma. (**D**) The inferior border often leaves a strip of overhanging bone, obscuring a clear operative trajectory toward the middle fossa floor. Subsequently, a rongeur or a handheld drill may be used to remove this overhanging bone until the edge of the craniotomy is at the level of the floor. The temporal bone and mastoid air cells are waxed to prevent development of a postoperative cerebrospinal fluid fistula.

**Figure 4:**
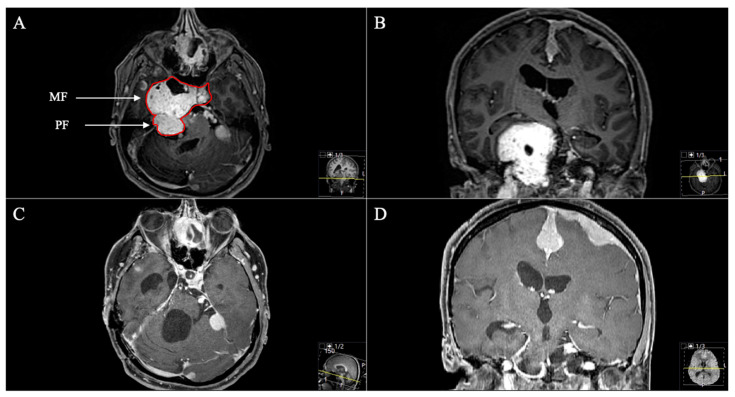
A 39-year-old male patient with neurofibromatosis type 2 presented with progressive vertigo, intermittent right facial pain and trigeminal hypesthesia (V1). (**A**) Preoperative axial and (**B**) coronal T1-weighted gadolinium-enhanced MRI, showing an impressive space-occupying cystic trigeminal schwannoma, involving the middle (MF) and posterior fossa (PF) through Meckel’s cave (Samii Type C). (**C**) Postoperative axial and (**D**) coronal MRI control, indicating complete resection via a two-stage technique; a modified Kawase and a retrosigmoid approach were performed. Postoperatively, no new deficits occurred and the patient recovered from the facial pain.

This group consists of the classic intradural subtemporal, a mainly extradural subtemporal and the Kawase approach; technical details have been described in detail previously [1,2,4,12,14,15,16]. Briefly, for an anterior transpetrosal approach, after a temporal craniotomy (Figure 3), the middle cranial fossa is exposed epidurally to the anterior portion of the petrous edge and the lateral edges of the oval and rotundum foramina. The middle meningeal artery (MMA) and the superficial greater petrosal nerve (GSPN) are important landmarks for these explorations. After cutting the MMA and detaching the GSPN, an anterior petrosectomy is performed. The tentorium is incised to obtain a wide operative field. The Meckel’s cave is opened. Tumors in the cave are usually wrapped in the plexiform portion of the trigeminal nerve. Although posterior fossa tumors sometimes develop more extensively than the approaching cavity, pulling the tumor toward its origin allows its removal. The posterior fossa tumors are usually adjacent to the fourth, sixth, seventh and eighth cranial nerves, but do not adhere to these nerves. Otherwise, a dural incision is performed over V3 and the oval foramen, the GSPN is identified from dorsal to anterior, the dura is further mobilized along the petrous ridge and the tumor is exposed intradurally. Alternatively, a C-shaped dura incision is performed immediately, cerebrospinal fluid is released and Meckel’s cave is exposed through a subtemporal approach (Figure 4).

#### 2.4.2. Retrosigmoid Approach

**Figure 5:**
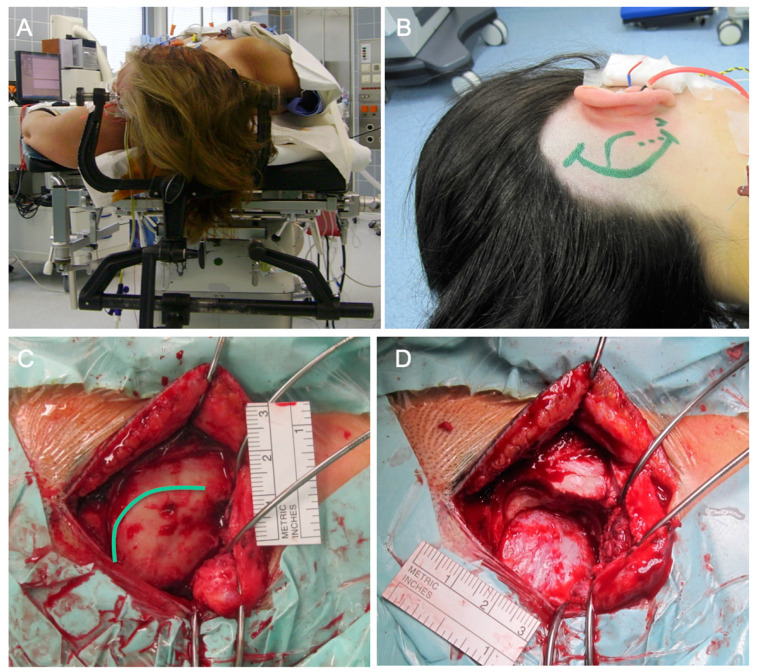
Intraoperative illustration of a retrosigmoid osteoclastic craniotomy. (**A**,**B**) The patient is placed in a supine position. The head should be rotated as much as possible (90°) while utilizing a large shoulder roll underneath the ipsilateral shoulder. A classic retroauricular curvilinear incision is performed. (**C**) Identification of the transverse-sigmoid junction will guide placement of a burr hole just below this junction. The burr hole is created where the transverse sinus (imaginary connection of inion with the root of the zygoma) crosses the sigmoid sinus at the level of the mastoid groove (green line). The asterion is not a constant finding and a burr hole over the asterion often exposes the entire width of the transverse sinus, placing this structure at risk. (**D**) The exact location of these dural sinuses is slightly variable and the initial burr hole should be placed with caution; it may be enlarged with further burr holes in the correct direction when the initial small pilot burr hole is done. The craniotomy may be continued by further osteoclastic enlargement with a rongeur or a handheld drill. Air cells are waxed to prevent development of a postoperative cerebrospinal fluid fistula.

**Figure 6:**
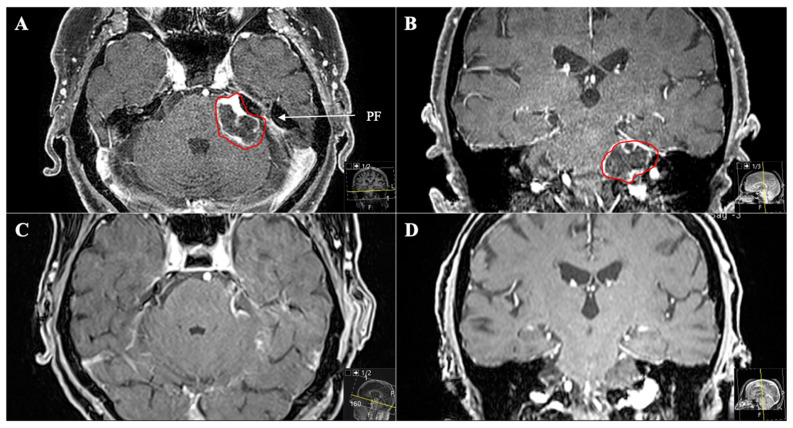
A 53-year-old female patient presented with vomiting, slight ataxia, facial pain and trigeminal hypesthesia (V1,V2) for three months. (**A**) Preoperative axial and (**B**) coronal T1-weighted gadolinium-enhanced MRI, showing a space-occupying cystic trigeminal schwannoma invading the cerebellopontine angle (Samii Type B). (**C**) Postoperative axial and (**D**) coronal MRI control, indicating complete resection via a retrosigmoid approach. Postoperatively, new temporary slight hypoacusis and facial neve palsy (House and Brackmann Grade III) occurred.

Analogue to the pterional approach for the anterior skull base, the retrosigmoid approach is the workhorse approach regarding the posterior fossa and the cerebellar-pontine angle [5,6,17,18]. We prefer a C-shaped skin incision. An osteoplastic or osteoclastic approach is performed, with the superior and anterior margins bordering the transverse and sigmoid sinuses, respectively (Figure 5). The dura mater is opened parallel to the sigmoid sinus; cerebrospinal fluid is drained from the cerebellomedullary cistern; cranial nerves VII–XI are identified. The tumour is exposed near the tentorium margin. After intracapsular tumor debulking, microsurgical radical removal is carried out (Figure 6).

#### 2.4.3. Transnasal Endoscopic (Transpterygoid) Approach

**Figure 7:**
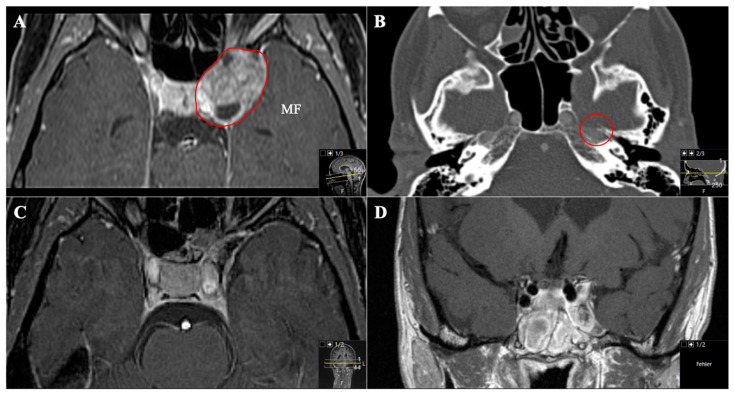
A 45-year-old female patient presented with left-sided visual impairment and headache. (**A**) Preoperative axial T1-weighted gadolinium-enhanced MRI and (**B**) axial CT, showing an optic-canal-compressing trigeminal schwannoma with a cystic part in Meckel’s cave in the middle fossa (MF, Samii Type A). Note how the foramen rotundum is widened (red circle). (**C**) Postoperative axial and (**D**) coronal MRI control, indicating complete resection via a transnasal endoscopic (transpterygoid) approach using a nasoseptal flap.

Endoscopic endonasal approaches can be used for trigeminal schwannomas restricted to Meckel’s cave and/or extracranial invasion [8,10]. In summary, we access the sphenopalatine foramen, uncinate process and the middle turbinate. For a sufficient transmaxillary corridor, an ethmoidectomy and sphenoidotomy may be needed as well. Finally, the sphenoid sinus is entered and enlarged. Neuronavigation is regularly used for better orientation and performing a tumour-targeting corridor. A transnasal endoscopic approach can be favored if the anatomical corridor is already widened by the given pathology as the small working space is therefore improved without extra preparation (Figure 7).

## 3. Results

### 3.1. Patient Population

Thirteen patients underwent resection for trigeminal schwannomas and were analyzed (patient characteristics see Table 1).

### 3.2. Tumour Location and Performed Approaches

Tumour locations were classified according to Samii’s and Ramina’s classification [5,6]. According to Samii et al. (Table 2), six schwannomas were classified as Type A, two as Type B, four as Type C and one as Type D. In two cases, the tumor was resected in a two-stage technique. The intradural subtemporal approach was performed in five cases, the extradural technique once, the retrosigmoid in five cases, the Kawase and a transnasal approach in two cases, respectively. The two cases with a two-stage approach (one neurofibromatosis type 2 (NF2) patient, Figure 1) were classified as Type E, according to Ramina et al., reflecting the surgical difficulty [6].

### 3.3. Postoperative Outcome

**Figure 8:**
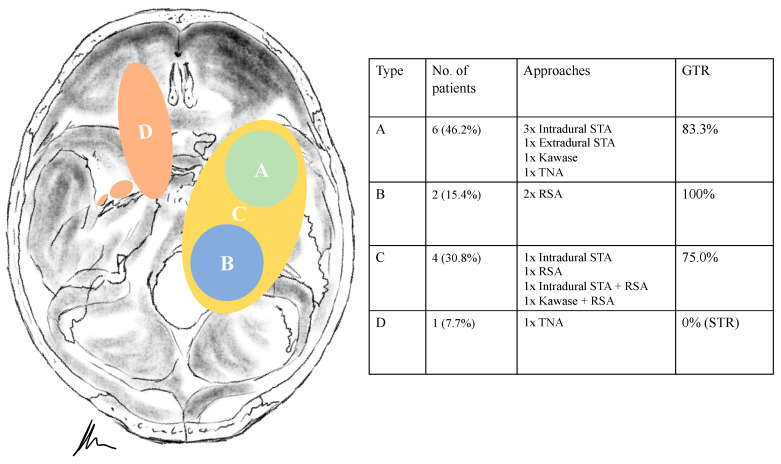
Illustration displaying surgical techniques and outcomes for each tumor type (according to Samii). STA = Subtemporal approach. TNA = Transnasal endoscopic approach. RSA = Retrosigmoid approach. GTR = Gross total resection. STR = Subtotal resection.

Median follow-up time was 24.0 (0–136) months. Follow-up MRIs have been performed in an annual fashion for five years and then enwidened to two-year intervals. All postoperative new permanent deficits were recorded during whole follow-up. GTR was achieved in 76.9% (10/13) (Figure 8). Three (23.1%) patients developed postoperative anesthesia in at least one branch of the trigeminal nerve, though one (7.7%) patient recovered during follow-up. It was possible to preserve trigeminal motor function in 11 (84.6%) patients. Two (15.4%) patients developed postoperative new oculomotor and abducens nerve palsy, respectively, but both recovered well during follow-up. Ten of the 12 patients (83.3%) who reported decreased gustation, cerebellar ataxia, vertigo, visual impairment, or headache improved postoperatively, but chronic headache in one patient remained. Preoperative hypoacusis did not improve as well. Two (15.4%) patients exhibited minimal facial palsy (House and Brackmann II-III) that resolved during the follow-up. One (7.7%) of the six patients who reported facial pain preoperatively remained symptomatic. 

**Figure 9:**
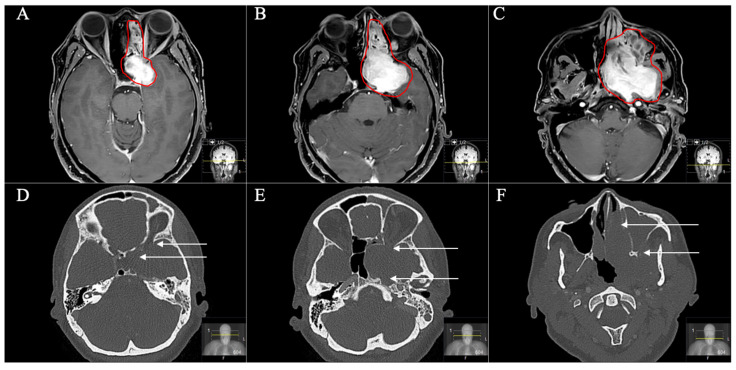
A 54-year-old female patient presented with slowly progressive visual impairment on the left side, trigeminal hypesthesia (V3) and an abducens nerve palsy with intermittent facial pain. (**A**–**C**) Preoperative axial T1-weighted gadolinium-enhanced MRI showing a massive space-occupying mainly extracranial trigeminal schwannoma (Type D) involving the middle fossa up to the orbital funnel. The schwannoma is bounded medially by the lamina papyracea, inferiorly by the hard palate and infiltrating the pterygopalatine fossa laterally. The middle nasal concha forms the anterior border. The paranasal sinuses are completely misplaced. (**D**–**F**) Preoperative axial CT scans demonstrating the destruction of the anterior skull base including the orbital funnel, the foramina ovale and rotundum and also the nasal septum and the maxillary sinus (arrows).

**Figure 10:**
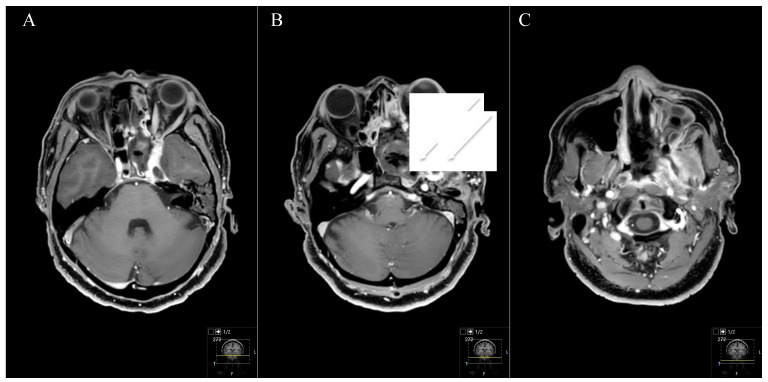
Postoperative outcome and MRI control (see Figure 9) (**A**–**C**) Postoperative axial T1-weighted gadolinium-enhanced MRI showing a satisfactory STR with small remnants in the maxillary sinus (arrows in (**B**)). Resection was performed by an endoscopic transnasal approach using the tumor-related widened corridor to the maxillary sinus. The patient recovered very well, the visual impairment recovered subsequently, the abducens nerve palsy and the facial pain during follow-up as well. The trigeminal hypesthesia remained. The patient had follow-up controls without any progress.

Two patients with STR underwent postoperative radiation with an uneventful course and stable disease. The patient with the Type D schwannoma had a transnasal approach with STR (Figure 9 and Figure 10). However, she showed no progress during follow-up. Total adverse event rate requiring surgical intervention during follow-up was 7.7%, with one patient developing a postoperative hydrocephalus during follow-up. The patient received a ventriculoperitoneal-shunt implantation. Surgery-related mortality was 0%.

## 4. Discussion 

In our series, good clinical outcome and satisfactory rates of GTR (76.9%) were achieved using classic and well-known skull-base approaches. Our findings are comparable with those of previous cohorts, but with fewer and technically more feasible approaches [1,4,5,6,10,16]. As good functional outcome with GTR can be achieved and deterioration is avoidable, we emphasize a surgical strategy to prevent cranial nerve injury, especially in case of huge schwannomas, as previously described [6,16,19].

Treatment strategy for trigeminal schwannomas should always respect the individual’s anatomy, clinical presentation and the patient’s baseline characteristics. As already described, diverse classifications have been proposed to facilitate decision making and assess the technical difficulty [3,4,6,11,20,21,22]. However, we believe the choice is mainly whether the tumor has to be approached via a posterior or a middle/anterior fossa approach. Thus, the basic division made by Samii et al. puts the options in a nutshell with following classification [5]: Type A, intracranial tumor predominantly in the middle fossa; Type B, intracranial tumor predominantly in the posterior fossa; Type C tumors in the middle and posterior fossa and Type D extracranial tumor with intracranial extensions.

Type A, C and D tumors can be targeted via a middle fossa approach, whereas Type B tumors are well managed through a retrosigmoid technique; in case of Type C tumors, a combined approach may be mandatory (Figure 1). Size of the tumor, goal of the surgery (biopsy, cranial nerve decompression, GTR) and the characteristics of the tumor must be considered. Type D tumors have a separate strategic position, as the extradural and extracranial portion of the schwannoma define the approach, ranging from an extradural subtemporal approach, an anterior/posterior petrosectomy to (endoscopic) transfacial or transnasal techniques [1,4,6,8,10,12,16].

Transnasal endoscopic approaches may be a minimally invasive technique in special conditions. It enables simple and atraumatic access to some Type A/D tumors. However, this technique has some limitations, such as minor exposure and surgical control of critical structures, a narrow surgical corridor and the risk of a cerebrospinal fluid leak [8,10]. Purely endoscopic surgery of large invading lesions requires substantial experience with this technique but satisfying results can be achieved. In this series, resection of a Type A and D schwannoma located close to the ethmoidal cells and maxillary sinus, respectively, was successfully performed (Figure 3).

Management of trigeminal schwannomas involves clinical and MRI-based follow-ups, surgical removal and, alternatively, radiotherapy or radiosurgery [2,6,12,23,24,25]. Microsurgical techniques have decreased the morbidity of trigeminal schwannoma surgery, but the risk of complications, such as cranial nerve palsy and cerebrospinal fluid leak, still challenges GTR. Those tumors seem to progress after STR [2]. Therefore, radiotherapy may be seen as a reasonable alternative to re-resection for such patients or for patients not suitable for resection generally. We think this technique can be reserved for small, nonresectable schwannomas or tumor remnants within the cavernous sinus. Median tumour volume was 8.05 cm^3^ and median maximal diameter 3.20 cm in our series. We observed that all patients were symptomatic at time of presentation and had relatively large space-occupying tumors. According to interdisciplinary tumour board discussions, primary surgical treatment was indicated first, also for histopathological safe diagnosis as trigeminal schwannomas only account for 0.1 to 0.5% of intracranial tumors [1,2]. We did not observe any incidental findings of a trigeminal schwannoma; all patients had a decrease in quality in life with also cranial nerve deficits (Table 1). Considering tumour volume and size, primary radiotherapy for tumor control may not be indicated at first. 

Stereotactic radiosurgery and fractionated radiotherapy have been performed for patients with intracranial schwannomas from different origins. Similar to an approach for vestibular schwannomas, treatment decisions should be made based on expected morbidity and tumour control. For larger lesions initial surgical resection followed by radiotherapy for residual tumor is an effective option. Larger tumour or tumour with brainstem compression should primarily be treated with surgery rather than radiosurgery [26]. However, if surgery is contraindicated, also for larger lesion, excellent tumor control rates at 5 and 10 years were achieved in 94% of patients with trigeminal schwannoma [27]. However, there appears to be an increased risk of transient enlargement and increased toxicity of large, cystic lesions undergoing radiotherapy [27].

For small vestibular schwannomas a high rate of tumour growth control (>95%) of five or more years after radiotherapy with lower morbidity compared to surgery can be expected [28,29]. Consequently, fractionated radiotherapy and stereotactic radiosurgery as an alternative to surgical resection for patients with smaller intracranial schwannomas, both vestibular and non-vestibular, are alternative treatment options. Retrospective analyses of patients treated with radiosurgery for jugular foramen tumors showed lower cranial injury after radiosurgery compared to surgery and favors radiosurgery as the preferred management strategy if patients do not have large tumors and symptomatic mass effect [30]. Tumor control rates for trigeminal schwannoma are excellent with both stereotactic radiosurgery and fractionated radiotherapy with minimal morbidity and toxicity [27]. The use of radiotherapy to treat trigeminal schwannoma resulted in functional improvement in 67.3% of patients, 26.9% had a stable lesion and worsening of the disease occurred in only two patients (3.8%) [31]. 

Radiotherapy in schwannomas is also well described regarding vestibular schwannomas in cases of NF2. Patients with NF2 play a special role, authors discussed optimal treatment strategies for especially vestibular schwannomas [2,32,33,34]. Nowadays, we know that each treatment decision in NF2 disease requires a complete evaluation of all cranial and spinal locations of the disease in order to establish surgical priorities and strategies. Yao et al. already stated in case of vestibular schwannomas, that preferred management is surgery followed by radiation, depending on institutional protocols [34]. They also reported that management for medium- or small-sized vestibular schwannomas may include wait and watch, radiotherapy and/or surgery regarding to the patients’ preference or also the institutional standards. Finally, they concluded that main purpose should be the preservation of cranial nerve function as long as possible in combination with tumor control in case of NF2. 

In a systematic review aimed to compare outcomes of surgery and stereotactic radiotherapy with no earlier intervention, pooled analysis demonstrated that stereotactic radiotherapy is superior to surgery in case of vestibular schwannomas with a diameter less than 3 cm. Both approaches were comparable in terms of tumor control. Stereotactic radiotherapy was associated with better facial nerve function, hearing preservation and quality of life. A cohort study compared the outcomes of stereotactic radiotherapy and surgery in vestibular schwannomas with a tumor diameter ≤ 2.8 cm with no differences observed in terms of functional outcomes, tumor control and mortality [34,35,36].

Despite of wide acceptance that radiotherapy is less effective in controlling NF2 vestibular schwannomas than sporadic ones, radiotherapy as a management option should still be discussed [34,37], but outcomes are varying in the literature so far. For example, Mathieu et al. have treated 62 of NF2 patients using radiosurgery. The mean tumor volume was 5.7 cm^3^ and serviceable hearing was present in 35%. The control and the hearing preservation rates were observed to be 85%, 81% and 81% at 5, 10 and 15 years and 73%, 59% and 48% at 1, 2 and 5 years, respectively. Tumor volume was significantly predictive of local control [38]. In another analysis, Phi et al. reported about 36 NF2 patients treated with radiosurgery with a mean tumor volume of 3.2 cm^3^. Five patients developed tumor recurrence and calculated control rates were 81%, 74% and 66% in the first, second and fifth year, respectively [34,39]. 

We surgically treated 175 vestibular schwannomas in the same time period, six cases had NF2 (4.0%), regarding trigeminal schwannomas only one of 13 patients (7.7%) had NF2 displaying the rarity of this special constellation [2,40,41,42]. Transferring the optimal treatment discussion to sporadic and NF2 trigeminal schwannomas, surgery should be considered in case of symptomatic ones, as it has been handled in our institutional tumour board. 

Patients with trigeminal schwannomas tend to present at a very late state, where cranial nerve compression or other tumour mass induced deficits has already occurred. With referring to tumour size and volume, taking optimal tumor size for stereotactic radiosurgery or gamma-knife in account, patients with trigeminal schwannomas tend not to be suitable for primary radiotherapy instead of primary surgical resection and debulking.

In addition, none of our patients received postoperative radiotherapy, extent of resection was sufficient and functional outcome satisfying. We believe that gross total resection of trigeminal schwannomas still remains superior to primary radiotherapy due to above-mentioned facts and as complete resection of benign schwannomas can be equated to cure (except in NF2). We advocate microsurgical complete resection, as several studies showed satisfying results in > 70% of the patients by means of skull-base approaches and microsurgical dissection [2,23]. We could show, that, in contrast to more complicated reported techniques, feasible and more basic skull base approaches may be sufficient enough to attack trigeminal schwannomas. Involvement of the cavernous sinus should be seen as one of the main causes of STR, but in a majority of cases, a clear plane of cleavage between the tumor capsule and the cavernous sinus structures can be identified, still enabling complete resection [6]. In a well-known series reported by Dolenc or Kawase [1,4,43], GTR was achieved in 100% and 74–82% of the patients, respectively, including ones who had undergone incomplete resection before and underwent re-resection with excellent outcomes, emphasizing the advantages of microsurgical therapy of trigeminal schwannomas. They recommended several extradural approaches to the schwannomas. We also experienced satisfying results with such techniques with good learning curves.

Based on our results, we think that more invasive or combined approaches are only needed if the tumor is extensively invading extracranial structures, or the anatomy is grossly altered; the transnasal approach is a simple and suitable option if indication is correctly based on the anatomic conditions. Nowadays, with superior preoperative visualization, a precise choice of approach can be made during presurgical planning. Most of the Meckel’s cave regions can be reached and overseen with different, yet standard, approaches, avoiding risk to the cavernous sinus, cranial nerve structures or visual apparatus. There is no superior or “one-size-fits-all” approach to trigeminal schwannomas, yet, based on our series, we think a “few-fits-most” concept of using standard approaches allows a very good outcome with a satisfactory learning curve in the majority of cases. With this concept of standardization, we believe more neurosurgical colleagues can achieve good surgical outcomes with these rare lesions.

### Study Limitations

As this was a retrospective case series, it was not possible to determine causalities with respect to clinical outcome. Nevertheless, we implemented detailed clinical examination, including scores on functional performance and a standardized follow-up protocol based on a certified neuro-oncological board in our clinical workflow. By focusing mainly on one entity, we aimed to avoid case heterogeneity—with a slight reduction of sample size.

## 5. Conclusions

Considering the low operative morbidity and satisfying functional outcome, complete resection of trigeminal schwannomas is possible. GTR is advocated, maintaining the functionality of cranial nerves by choosing well-tailored and if needed, combined approaches. In case of STR, postoperative radiation may be discussed for better local control. The existing classification systems should be seen as an additive armamentarium for preoperative planning. A transnasal endoscopic technique must be regarded with caution due to its limitations. Classic and standardized skull-base approaches are advocated and can be combined to avoid more difficult and invasive approaches with higher perioperative morbidity.

## Figures and Tables

**Table 1:** Demographics, clinical presentation and tumour histopathology. Data shown as n = number (%). Mdn. = median [interquartile range].

Mdn. Age (years)		57.5	(36–83)
Sex	Female	7	(53.8%)
	Male	6	(46.2%)
Preoperative Deficits			
Decreased gustation		2	(15.4%)
Cerebellar ataxia		2	(15.4%)
Vertigo/Nausea		2	(15.4%)
Headache		3	(23.1%)
Visual impairment		3	(23.1%)
Hypacusis		4	(30.8%)
Facial pain		7	(53.8%)
Trigeminal nerve hypesthesia			
V1		6	(46.2%)
V2		8	(61.5%)
V3		7	(53.8%)
Mdn. duration of symptoms (months)		6	(0–288)
Histopathology			
Schwannoma WHO grade I		13	(100%)
Mdn. Tumour Volume (cm^3^)		8.05	(2.7–81.8)
Mdn. max. Tumour Diameter (cm)		3.20	(2.6–7.2)
Mean max. Tumour Diameter (cm)		3.44	

**Table 2:** Types of tumors and surgical approaches. Data shown as n = number (%). STA = Subtemporal approach. TNA = Transnasal endoscopic approach. RSA = Retrosigmoid approach.

Type of Tumour	Samii et al.	Ramina et al.	No. of Patients	Approach	
	A	C	6 (46.2%)	Intradural STA Extradural STA Kawase TNA	3 (23.1%) 1 (7.7%) 1 (7.7%) 1 (7.7%)
	B	D	2 (15.4%)	RSA	2 (15.4%)
	C	E	4 (30.8%)	Intradural STA	1 (7.7%)
				RSA Intradural STA + RSA	2 (15.4%) 1 (7.7%)
				Kawase + RSA	1 (7.7%)
	D	A	1 (7.7%)	TNA	1 (7.7%)

## Data Availability

The data presented in this study are available on request from the corresponding author.
